# Carcinoma *in situ* of true vocal cord in a nonsmoker adolescent female

**DOI:** 10.4103/0971-5851.56337

**Published:** 2009

**Authors:** Dodul Mondal, Sayan Kundu, Subrata Chattopadhyay, Sumitava De, A Ghosh Dastidar, Amitabha Roy

**Affiliations:** *Department of Radiotherapy, Medical College Hospital, Kolkata, India*

**Keywords:** *Carcinoma in situ*, *true vocal cord*, *adolescent*, *non smoker female*

## Abstract

Carcinoma *in situ* (precancerous lesion) of true vocal cord in a nonsmoker adolescent female without any history of prior neck irradiation is rare. A 16-year-old female patient without any of the known risk factors presented with history of gradual-onset hoarseness of voice unrelieved by symptomatic treatments for 1 year. Contrast-enhanced CT scan of neck and laryngoscopy and histopathology of the tissue from irregular lesions along the medial margin of the left vocal cord diagnosed it as a case of carcinoma *in situ* of vocal cord. Absence of known risk factors and very young age of the patient made this case a rarity and hence the case is being reported.

## INTRODUCTION

Vocal cord cancer is usually a disease of the middle-aged chronic smoker, being more common among males (M: F = 3:1). Prior neck irradiation for some other disease is also a risk factor. Glottic cancer is more common than supraglottic cancer. Heavy smoking and adult age are the main known risk factors.

## CASE REPORT

**Figure 1:**
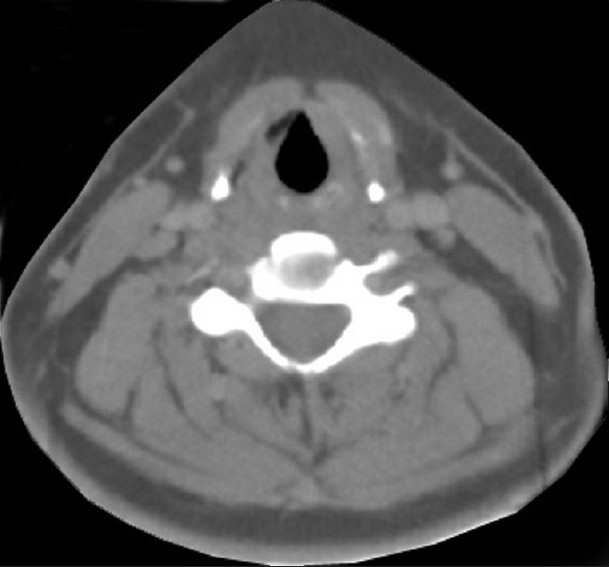
CECT scan of neck showing irregular lesion along the medial margin of left vocal cord at the junction of anterior one third and posterior two thirds. There is thickening of left true vocal cord with loss of paraglottic fat, suggestive of a neoplastic lesion. The normal paraglottic fat on right side is seen as black area just deep to the thyroid cartilage (seen as white). The airway is seen as black oval structure

**Figure 2:**
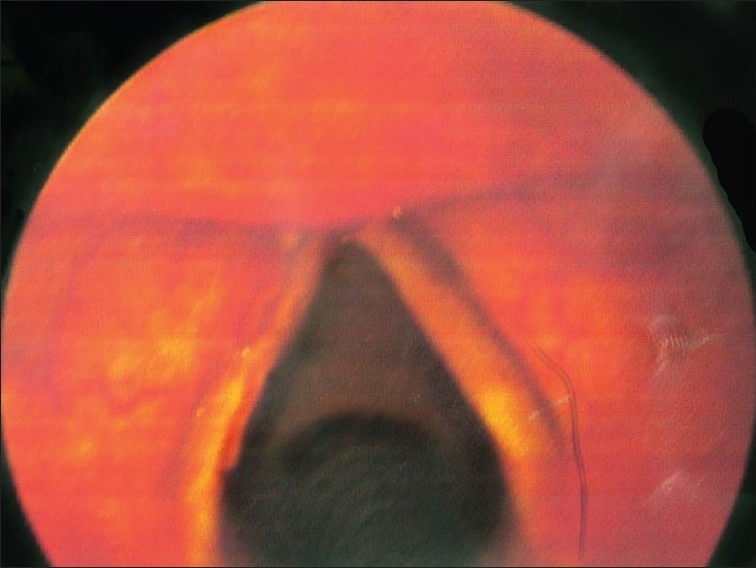
Fiber-optic laryngoscopy showing irregular lesion along the medial margin of left vocal cord in the anterior part

**Figure 3:**
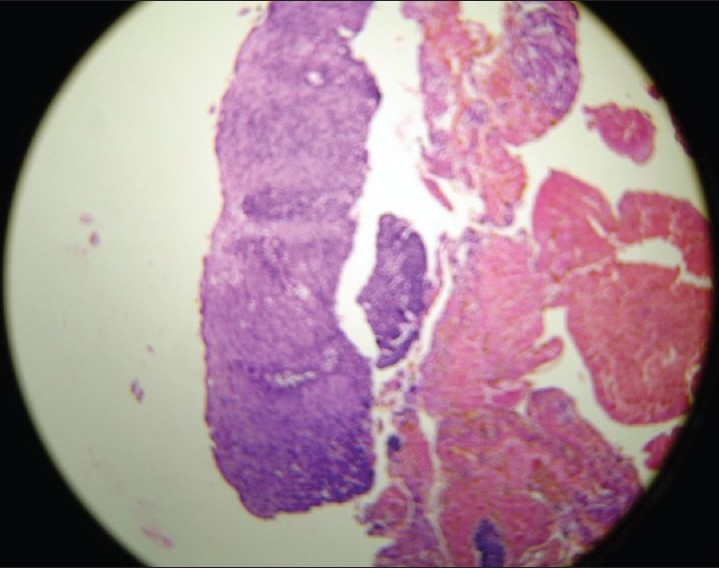
Histopathological slide of left vocal cord lesion in low-power field showing full-thickness severe dysplastic changes of epithelium

**Figure 4:**
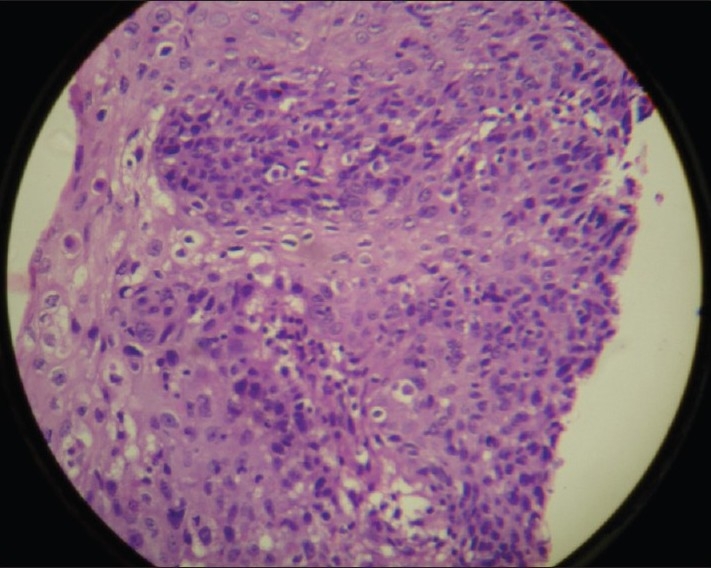
High-power field of the same histopathological slide [Figure 3], showing a more detailed view

A 16-year-old female patient presented to the radiotherapy department in July 2008 with complaint of unrelenting hoarseness of voice for the last 1 year. She developed it gradually over a period of time. Initial treatment was directed to common cold but was of no help. There was no associated sore throat, otalgia, localized pain or tenderness over thyroid cartilage, dysphagia or features of airway obstruction. The patient is a nonsmoker and is nonalcoholic and without any history of past or present marijuana smoking. There was no history of prior neck irradiation. An incidence of cancer in the family could not be elicited from a detailed history. An indirect laryngoscopy was performed, which showed both vocal cords to be mobile. There was no visible growth over any of the vocal cords. Contrast-enhanced CT scan of the neck was performed, and it showed a small irregular lesion over the left vocal cord at the junction of anterior one third and posterior two thirds [Figure 1]. Thickening of left true vocal cord with loss of paraglottic fat is seen, suggestive of a neoplastic lesion. The normal paraglottic fat on the right side is seen as black area just deep to the thyroid cartilage (seen as white). The airway is seen as black oval structure. There is no visible neck node. Fiber-optic laryngoscopy confirmed the CT scan finding, which also showed irregular lesions along the medial margin of the left vocal cord [Figure 2]. Both CT scan and fiber-optic laryngoscopy showed an otherwise normal picture. Microlaryngoscopic removal of the mass was performed. Histopathological examination under low-power and high-power fields showed severe dysplasia and full-thickness replacement of epithelium with dysplastic cells and diagnosed it as carcinoma *in situ* [Figures 3 and 4]. However, the risk of a micro-invasive carcinoma could not be ruled out. A second biopsy was advised to obtain deeper tissue, but it was refused by the patient. So from the available documents, we considered it carcinoma *in situ* of the vocal cord.

A detailed physical examination was performed, which was unremarkable. There was no palpable lymph node in neck and other sites. Complete hemogram, liver function test, chest X-ray, renal function test and ultrasonography of the whole abdomen were performed as part of routine investigations before commencing therapy, all of which were unremarkable. The stage of the disease was Tis N0 M0.

After reaching at a specific diagnosis and doing all pretreatment workup, specific anticancer therapy was started with radical radiotherapy (60 Gy in 30 fractions, 2 Gy per fraction, 5 days a week for 6 weeks) using Telecobalt (Theratron 780 C) machine. Radiotherapy was started with two lateral opposed fields to neck to include the vocal cord (a 5 × 5 cm field) extending from superior border of thyroid cartilage superiorly to lower border of cricoid cartilage inferiorly sparing the spinal cord and other organs at risk. A CT-based treatment planning was done to achieve maximum dose to vocal cord, sparing normal tissue as much as possible.

## DISCUSSION

Laryngeal cancer is a rare entity among children[1] and young population.[2] A total number of 24,960 cancers were found to be diagnosed in children younger than 19 years through the Survival, Epidemiology and End Results (SEER) database from 1973 through 1996. Among this population, 3050 (12%) tumors were located in the head and neck. Incidence of pediatric squamous cell carcinoma was rare (2%) among these populations, and laryngeal carcinoma was even more rare.[3] Laryngeal carcinoma is one of the malignancies that are diagnosed at an early stage due to production of symptoms at a very early stage in the form of change of voice that does not respond to common treatments. Carcinoma *in situ* is common in vocal cord. Distinction between carcinoma *in situ*, micro-invasive squamous cell carcinoma and invasive carcinoma may be troublesome in many cases. The usual patient with laryngeal cancer is a middle-aged male chronic smoker. At presentation about 51% are localized to larynx, 29% have regional spread and 15% have distant metastasis.[4] Cigarette smoking and tobacco use are well-known risk factors. The role of alcohol is unclear.[5] There is some evidence to suggest that heavy marijuana smoking may be a risk factor for development of laryngeal cancer in young individuals.[6] Prior irradiation to neck for some other condition is also a risk factor. Most carcinomas of true vocal cord arise at the anterior two thirds. Majority of them start in the free margin of the vocal cord and at the upper surface.

It should be noted that our case is unique in the sense that all these known risk factors were absent in this patient.

Squamous cell carcinoma is the major histopathological type. Less frequent pathological subtypes are spindle cell carcinoma, verrucous carcinoma, neuroendocrine tumor and minor salivary gland tumor.[7] There are reports of cases of laryngeal carcinoma of clear cell type[8] and adenoid cystic type[9] in children.

Hoarseness of voice not responding to treatment for common conditions is the most common presenting symptom for most of the early-stage vocal cord cancers. Advanced cancers may present with sore throat, otalgia, localized pain and tenderness over thyroid cartilage; or sometimes, dysphagia. If the tumor is bulky or large enough, it can produce symptoms and signs of airway obstruction. Incidence of clinically positive neck nodes varies according to the tumor stage: T1, approximately 0; T2, ≤ 2%; T3, T4, 20%-30%.[10] 

Contrast-enhanced CT scan of the neck is the investigation of choice[11] for laryngeal malignancy and is preferred over MRI, as the latter takes more time; and as a result, risks of motion artifact are more for MRI.[12] CT scan is almost always done before taking biopsy specimen to avoid confusion between a malignant lesion and post-biopsy changes of the cord. CT scan is also useful to detect subglottic extension of vocal cord lesions and also for CT-based planning for radiotherapy.

Patients diagnosed as having carcinoma *in situ* of true vocal cord may be treated by stripping of the lesion, CO_2_ laser excision and early irradiation. However, early irradiation often provides better voice preservation[13] and eliminates the possibility of multiple recurrences and multiple biopsy procedures, which are very common for stripping. The possibility of invasive carcinoma should be in mind, as the risk of invasive component is always there in patients with carcinoma *in situ*.

## CONCLUSION

It may be stated that vocal cord carcinoma in a 16-year-old female without any risk factor is a rare entity, and clinicians should be aware of the possibility of this condition. Aggressive treatment has a chance of better outcome. Delay in suspecting, diagnosing and treating this disease may result in decreased cure rate.
